# Application of Solid Phase Extraction on Multiwalled Carbon Nanotubes of Some Heavy Metal Ions to Analysis of Skin Whitening Cosmetics Using ICP-AES

**DOI:** 10.3390/ijerph10010361

**Published:** 2013-01-14

**Authors:** Ayoub A. ALqadami, Mohammad Abulhassan Abdalla, Zeid A. ALOthman, Kamal Omer

**Affiliations:** Department of Chemistry, College of Science, King Saud University, P.O. Box 2455, Riyadh 11451, Saudi Arabia; E-Mails: aayoub17@yahoo.com (A.A.A.); zaothman@ksu.edu.sa (Z.A.A.); kmohmmed@ksu.edu.sa (K.O.)

**Keywords:** cosmetics, heavy metals, ICP-AES, preconcentration, solid phase extraction, MWCNT

## Abstract

A novel and highly sensitive method for the determination of some heavy metals in skin whitening cosmetics creams using multiwalled carbon nanotubes MWCNTs as solid phase extraction sorbent for the preconcentration of these heavy metals prior to their determination by inductively coupled plasma atomic emission spectrometry is described. Different practical parameters have been thoroughly investigated and the optimum experimental conditions were employed. The developed method was then applied for the determination of arsenic, bismuth, cadmium, mercury, lead and titanium in samples of skin whitening cosmetics. The detection limits under these conditions for As, Bi, Cd, Pb, Hg and Ti were 2.4, 4.08, 0.3, 2.1, 1.8, and 1.8 ng·mL^−1^, respectively. The relative standard deviations (RSDs) were found to be less than 2.0%. For validation, a certified reference material of NIST SRM 1570a spinach leaves was analyzed and the determined values were in good agreement with the certified values. The recoveries for spiked samples were found to be in the range of 89.6–104.4%.

## 1. Introduction

The appearance of spots on the skin is a source of concern for many people, especially women. These spots are caused by skin disorders or the existence of an excessive amount of melanin produced by melanocytes responsible for the pigmentation of the skin. This may occur for a variety of reasons, including excessive exposure to solar radiation, aging, weak hormones during pregnancy or by ingestion of certain drugs [1]. The disorder can be reduced through the use of whitening products, although the most serious cases require medical assistance. These products contain various chemicals such as kojic dipalmitate (KDP), which works as a whitening agent on the skin, based on different mechanisms [2]. Unfortunately, some of skin whitening products contain heavy metals such as mercury which can be absorbed through the skin and can cause deleterious effects in the body [3,4].

The mercury content of cosmetics was analyzed by Uram *et al.*, who found that the mercury concentration of skin whitening cosmetics was between 0.13 and 7.5 µg·g^−1^ [5]. Al-Saleh *et al.* analyzed some types of whitening creams from different countries, some of them containing high concentrations of mercury. In that study, the analyzed facial creams produced in England and Thailand contained the highest levels of mercury, ranging from 1,281 to 5,650 µg·g^−1^ [6]. Heavy metals like Pb, Cd, Hg, As and trace elements in 21 herbal cosmetic preparations sold in Indian market were analyzed by Sukender *et al.* They found that the results indicated that among the toxic heavy metals, two samples for Hg content and six for Pb content exceeded the WHO permissible limits fixed for herbal preparations and arsenic was found appreciably well below the permissible limit, but Cd was found above the permissible limit in the all samples [7].

In this study a novel and highly sensitive method using a microcolumn packed with MWCNTs for preconcentration of some heavy metals in skin whitening cosmetics prior to their determination by inductively coupled plasma atomic emission spectrometry is described.

## 2. Materials and Methods

### 2.1. Instrument

Inductively coupled plasma atomic emission spectroscopy (Thermo Scientific model ICAP 6000 series, Cambridge, UK) was used for determination of analytes. The optimum operation conditions for ICP-AES are summarized in Table 1. The pH values of solution were controlled with a pH meter (Thermo Orion 5 star Corporation, Beverly, MA, USA) supplied with a combined electrode. A closed-vessel microwave digestion system (Ethos-1600, Milestone, Bergamo, Italy) equipped with fiber optic temperature and pressure sensors were used for sample digestion. A self-made polytetrafluoroethylene (PTFE) microcolumn of 55 mm in length with an inner diameter 2.0 mm packed with 35 mg of multiwalled carbon nanotubes was used for the preconcentration/separation process. It was coupled to a peristaltic pump (Ismatec model ISM834c, Wertheim, Germany).

### 2.2. Reagent and Standard Solution

All chemicals used in this study, were of analytical reagent grade. The water used for all dilutions in all our experimental was of high purity (Milli-Q Millipore 18.2 MΩ·cm^−1^ conductivity). Stock standard solutions (1,000 μg·mL^−1^) for As, Bi, Cd, Hg, Pb and Ti were obtained from Spex Industries Inc. (CertiPrep, NJ, USA) Nitric acid 69–71% was purchased from Loba Chemie. Ltd. (Mumbai, India). Hydrogen peroxide solution 35% v/v. was from Merck (Darmstadt, Germany). Hydrofluoric acid 40% was purchased from Merck (Mumbai, India). The following buffer solutions were used to control the pH of the solutions: H_3_PO_4_-NaH_2_PO_2_H_2_O (pH 2), NaH_2_PO_4_-NaOH (pH 3–8), NH_4_Cl-NH_3_ (pH 8–10) and were purchased from BDH (Poole, UK). A standard reference material, NIST SRM 1570a Spinach leaves, was obtained from National Institute of Standards and Technology (NIST, Gaithersburg, MD, USA). Multiwalled carbon nanotubes MWCNTs with purity more than 95% and density 2.1 g/cm^3^ at 20 °C, was purchased from Chengdu Alpha Nanotechnology Ltd., (Taiwan, China). All the plastic and glassware were cleaned by soaking in dilute HNO_3_ 5% and were rinsed with distilled water before were use.

**Table 1:** Operation condition parameters for ICP-AES.

RF generator power (w)	1,150 W
Coolant gas flow rate	12 L·min^−1^
Auxiliary gas	0.5 L·min^−1^
Pump rate	25 rpm
Plasma view	Axial
Number of measurements	3
Analytical Wavelengths (nm)	As (189.00); Bi (223.00)
	Cd (228.80 ); Pb (220.30)
	Hg (184.95); Ti (334.90)

### 2.3. Collection of Samples

The samples were collected from various retail shops, pharmacies and beauty aid stores in the local market of Saudi Arabia. A total of 34 skin-whitening cosmetics were analyzed for determination of heavy metals such as As, Bi, Cd, Hg, Pb and Ti. The skin whitening cosmetics were imported from different countries such as China, Thailand, *etc.*

### 2.4. Preparation of Microcolumn

The microcolumn was prepared by placing 35 mg of the MWCNTs into an empty PTFE microcolumn (55 mm length × 2.0 mm i.d.). A small portion of glass wool was plugged at both ends of the microcolumn in order to avoid any loss of MWCNTs during the washing/preconcentration and elution steps. To form preconcentration system, the microcolumn was connected to a peristaltic pump with tubing. Before use, 10 mL of 1 mol·L^−1^ HNO_3 _solution and 30 mL of high purity deionized water were passed through the column in order to clean and condition it. Then, the column was conditioned to pH 7.5 with 2.0 mL of buffer solutions.

### 2.5. Sample Preparations

The majority of the analytical techniques used for determining heavy metals in cosmetic samples require the dissolution and dilution of samples in an appropriate solvent. However, most cosmetic samples require a pretreatment like complete acid digestion with microwave energy that permits rapid heating of samples, which considerably reduces the pretreatment time.

#### 2.5.1. Preparations of Standard Solution

From 1,000 µg·mL^−1^ stock standard solutions of As, Bi, Cd, Hg, Pb and Ti more dilute standard solutions ware prepared by further dilution of these solutions.

#### 2.5.2. Application to Microwave Digested Samples

Accurately weighed samples (0.1–0.25 g) were transferred to a 120 mL Teflon digestion vessel avoiding contact with the side of the vessel. Conc. nitric acid (5.0 mL) was added, followed by hydrogen peroxide (35%, 2.0 mL) and hydrofluoric acid (40%, 1.0 mL) added to the vessel using a graduated pipette. The vessel was sealed and left for about 15 minutes to ensure complete reaction. The sample was digested in a microwave (Milestone Ethos 1600) following the heating program shown in Table 2. After cooling to room temperature, the vessel was unsealed and the inner wall and lid were thoroughly rinsed with deionized water and deionized water (20 mL) was added to the digested solution. The solution was filtered through Whatman paper No.1 into a 50 mL polypropylene volumetric flask and diluted to volume with deionized water. Then, the sample was transferred to polypropylene bottles.

**Table 2:** Microwave heating program.

Step	(min) Time	(W) Power	Temp. (°C)
1	15	450	195
2	2.0	0	190
3	10	300	195
4	15	350	195

#### 2.5.3. General Preconcentration Procedure

After digestion of a sample, the sample solution was adjusted to the desired pH of 7.5 with buffer solution before use. Approximately 53 mL of prepared sample solution was passed through the microcolumn with a peristaltic pump at a flow rate of 2.0 mL·min^−1^. The retained ions were eluted from the column by 2.0 mL of 1.0 mol·L^−1^ HNO_3_ solution and 6.0 mL of deionized water at a flow rate of 2.0 mL·min^−1^, respectively. Then, the eluted heavy metal ions were analyzed by ICP-AES. The solution blank was prepared under the same conditions as in sample preparation but without adding the sample.

## 3. Results and Discussion

In order to obtain quantitative recoveries of the metal ions on multiwalled carbon nanotubes, the separation procedure was optimized for various analytical parameters such as pH, sample flow rate and optimization of elution conditions.

### 3.1. Effect of pH on Adsorption

**Figure 1:**
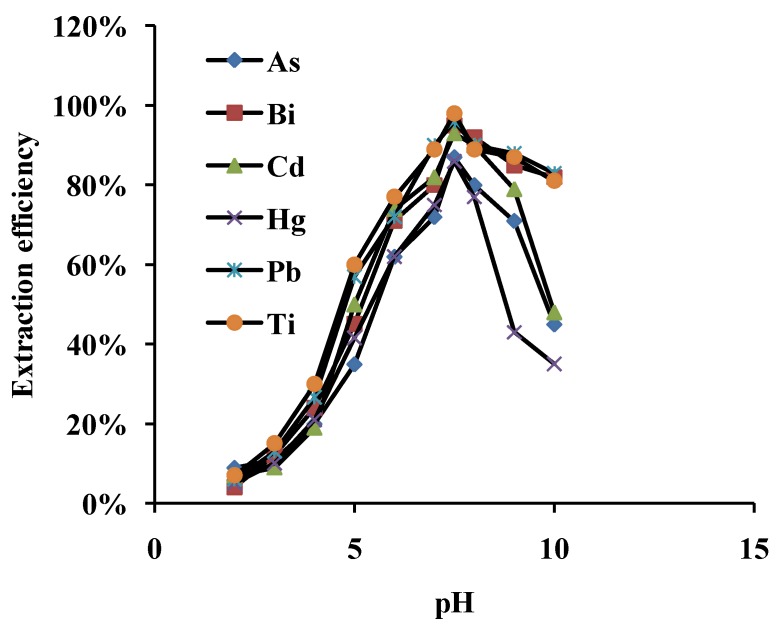
Effect of pH on the adsorption of As, Bi, Cd, Hg, Pb and Ti onto MWCNTs. Concentration of studied ions: 0.1 µg·mL^−1^ (N = 3).

The pH of an aqueous sample is a very important factor in the extraction efficiency of metal ions in solid phase extraction studies [8,9,10,11], hence, the effect of pH on the adsorption of As, Bi, Cd, Hg, Pb and Ti onto MWCNTs was investigated in the pH range of 2.0–10.0 by use of standard solutions containing 0.1 μg·mL^−1^ for each element keeping other parameters constant and adjusting with buffer solutions in order to obtain the optimal pH for the retention of each analyte ion. The extraction efficiencies were calculated from the difference between the spiking concentration and actual one measured. We observed that the extraction efficiency values decreased beyond pH 7.5, due to the fact that an increase of pH higher than 7.5 lead to the precipitation of the heavy metals. The surface isoelectric point of MWCNTs shifts to lower pH values upon neutralization of the MWCNTs’ surfaces, so cation adsorption decreases quickly [12]. The effect of pH values on the extraction efficiency is shown in Figure 1. It was seen that the optimum pH for extraction efficiency (>90%) of metal ions was pH 7.5, so all further work was performed at pH 7.5.

### 3.2. Effect of Sample Flow Rate

**Figure 2:**
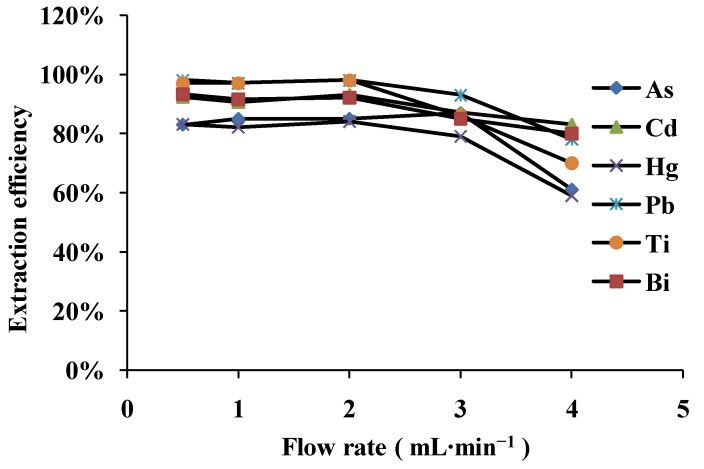
Effect of sample flow rate on extraction efficiency of the analytes As, Bi, Cd, Hg, Pb and Ti onto MWCNTs: 0.1 µg·mL^−1^; sample volume: 20 mL (N = 3).

The sample flow rate through the microcolumn filled with MWCNTs is a very important parameter, because it affects the retention of metal ions on the MWCNTs and the duration of the complete analysis. Therefore, the effect of the sample flow rate on extraction efficiency of As, Bi, Cd, Hg, Pb and Ti on multiwalled carbon nanotubes was investigated in the range (0.5–4.0 mL·min^−1^) by passing 25 mL of sample solution during the microcolumn with a peristaltic pump. The result is shown in Figure 2. No clear effect on analyte extraction efficiency was seen in the range 0.5–2.0 mL·min^−1^, but the extraction efficiency decreased over 2.0 mL·min^−1^ because an increase of the sample flow rate leads to lower metal adsorption on the MWCNTs, so 2.0 mL·min^−1^ was chosen as the flow rate of the sample solutions in subsequent experiments.

### 3.3. The Optimization of Elution Conditions

#### 3.3.1. Type of Eluent

Hydrochloric and nitric acid were used as eluents for the desorption of heavy metal ions from the multiwalled carbon nanotube column. The results for this experiment are given in Table 3. They show that extraction efficiency for the analyte ions using nitric acid as eluent is higher than with hydrochloric acid. The extraction efficiency values for all analyte ions were higher than 90% with 1.0 mol·L^−1^ HNO_3_. Thus, HNO_3_ had a better elution performance due to its oxidative action and stronger dissolution ability than HCl.

**Table 3:** Effect of type eluent.

Type of eluent	Extraction efficiency%					
	As	Bi	Cd	Hg	Pb	Ti
HCl 0.5 mol·L^−1^	20.2	25.4	18.1	13.2	21.5	31
HNO_3_ 0.5 mol·L^−1^	88.0	90.0	78.0	65.3	73.3	83
HCl 1.0 mol·L^−1^	29.2	33.0	35.0	29.0	40.8	85
HNO_3_ 1.0 mol·L^−1^	92.0	103	93.3	90.1	96.3	95

#### 3.3.2. Concentration of Eluent and Volume

**Figure 3:**
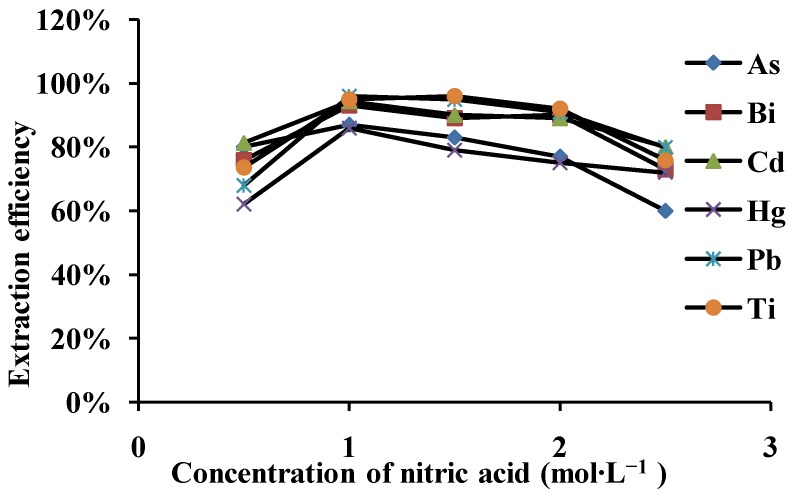
Effect of nitric acid at various concentrations as eluent on extraction efficiency of the metal ions (N = 3).

After nitric acid was chosen as eluent, and because of the adsorption of heavy metal ions at pH < 2 is negligible, for this reason, 2.0 mL of various concentrations (0.5–2.5 mol·L^−1^) of HNO_3_ were studied for the desorption of the retained analytes from the microcolumn. Figure 3 shows the effect of various concentrations of nitric acid on the desorption of 0.1 μg·mL^−1^ As, Bi, Cd, Hg, Pb and Ti. It was found that 1.0 mol·L^−1^ HNO_3_ was sufficient for quantitative elution (>91%).

The effect of eluent volume on the extraction efficiency of the analytes was also studied by keeping the HNO_3 _concentration constant at 1.0 mol·L^−1^. Effect of eluent volume values on the recoveries is shown in Table 4. It was found that the highest extraction efficiency (>90%) could be obtained with the use of 2.0 mL of 1.0 mol·L^−1^ HNO_3_, which sufficient for quantitative elution. Thus, the eluent concentration of 1.0 mol·L^−1^ HNO_3_ and elution volume of 2.0 mL was used in the following experiments.

**Table 4:** The effect of eluent volume on the recoveries of the analytes.

Volume of HNO_3 _eluent	Extraction efficiency					
	As	Bi	Cd	Hg	Pb	Ti
1 mL	87.0	83.0	90.0	88.0	90.8	91.0
2 mL	92.0	103	93.3	90.1	96.3	95.0
3 mL	92.6	97.0	92.0	93.0	93.6	95.0
4 mL	92.3	98.5	91.8	89.7	93.0	94.2

#### 3.3.3. Flow Rate

The effect of flow rate of eluent solution of 1.0 mol·L^−1^ HNO_3_ on the desorption of As, Bi, Cd, Hg, Pb, and Ti ions from the multiwalled carbon nanotube column was investigated in the range 0.5–4.0 mL·min^−1^ keeping other conditions constant. It was found that the best extraction efficiency could be obtained with the elution flow rate varying between 0.5–2.0 mL·min^−1^. Therefore, a flow rate of 2.0 mL·min^−1^ is chosen as optimum.

### 3.4. Adsorption Capacity

**Figure 4:**
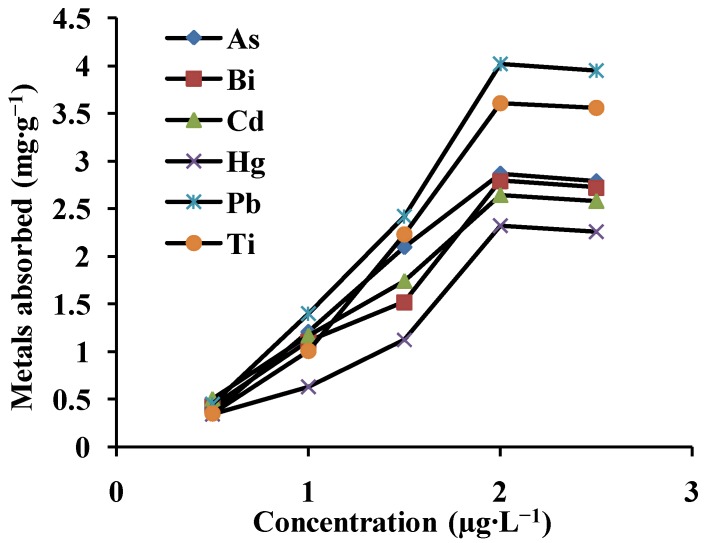
The breakthrough curves of the metal ions on MWCNTs at pH: 7.5; sample volume: 25 mL (N = 3).

Adsorption capacity is an important parameter for the evaluation of an adsorbent because it determines how much sorbent is required to quantitatively concentrate the analytes from a given solution. The adsorption capacity was investigated by a method provided in the literature [9,13]. For this purpose, a 25 mL aliquot of a series of concentrations (0.5–2.5 μg·mL^−1^) was adjusted to the appropriate pH, then preconcentrated and eluted. The amount of metal ions adsorbed at each concentration level was determined by ICP-AES. The breakthrough curves were obtained by plotting the metal ion concentrations in µg·mL^−1^ *versus* the milligrams of metal ions adsorbed per gram of adsorbent (Figure 4). It is seen from Figure 4 that the capacity of MWCNTs for metal ions was found to be 2.87, 2.79, 2.64, 2.32, 4.02, and 3.61 mg·g^−1^ for As, Bi, Cd, Hg, Pb, and Ti, respectively. We conclude that the adsorption of the multiwalled carbon nanotubes was excellent.

### 3.5. Column Reuse

In order to examine the long-term stability of MWCNTs, the column was subjected to successive adsorption and desorption cycles by passing 20 mL of the solutions containing the analytes through the column. The stability and potential regeneration of the column was assessed by monitoring the changes in the recoveries of the analytes. The column can be reused after regeneration with 2.0 mL of 1.0 mol·L^−1^ HNO_3_ and 30 mL of deionized water, respectively, and was stable up to 35 adsorption elution cycles without obvious decrease in the adsorption capacity or the recoveries of the analytes.

### 3.6. Applications of the Presented Procedure

The accuracy of the results for the presented study was demonstrated by analyzing the standard reference material NIST SRM spinach leaves 1570a after microwave digestion. The results are given in Table 5. The relative standard deviation of the proposed method in the range 2.5–12.8% was compared to the relative standard deviation of the certified value for NIST SRM 1570a in the range 2.42–17.64%. The results are in a good agreement with the certified values for the analytes.

**Table 5:** The results for reference standard materials (N= 3).

Element	NIST SRM 1570a Spinach leaves (mg·kg^−1^) ^a^	
	Certified value	Our value
As	0.068 ± 0.012	0.070 ± 0.009
Cd	2.89 ± 0.070	2.790 ± 0. 1
Hg	0.030 ± 0.003	0.028 ± 0.007
Pb	0.2	0.192 ± 0.02

^a^ Mean value ± standard deviation; N = 3.

### 3.7. Performance of the Presented Procedure

The accuracy of the results for the study was demonstrated by analyzing spiked concentrations of analyte ions after addition of known amounts of analytes into 25 mL of cosmetic samples. The results are given in Table 6.

**Table 6:** Addition/recovery test as the application of the presented method (N = 3).

Metal	Added	Yang Chin sample		Magical mix sample		Nivea Lotion sample	
	(μg)	Found (μg)	Recovery (%)	Found (μg)	Recovery (%)	Found (μg)	Recovery (%)
As	0	0.987	-	0.810	-	0.825	-
	2.5	3.250	90.5	3.110	92.0	3.090	90.6
Bi	0	BDL	-	26.630	-	0.412	-
	2.5	2.610	104.4	29.200	103.0	2.920	100.3
Cd	0	0.218	-	0.041	-	0.055	-
	2.5	2.510	91.7	2.600	102.0	2.310	90.2
Hg	0	BDL	-	41.720	-	1.200	-
	2.5	2.250	90.0	43.980	90.4	3.440	89.6
Pb	0	23.880	-	21.370	-	77.360	-
	2.5	26.200	93.0	23.650	91.2	79.950	103.6
Ti	0	16.080	-	72.890	-	0.431	-
	2.5	18.450	95.0	75.300	96.4	2.900	98.8

#### 3.7.1. Detection Limits and Precision

The detection limits of this method for As, Bi, Cd, Hg, Pb and Ti, were calculated under the optimized conditions after application of the preconcentration procedure to blank solutions. Based on three times the standard deviation of eight runs of the blank solution, they were found to be 2.4, 1.08, 0.3, 2.1, 1.8, and 1.8 ng·mL^−1^, respectively. The relative standard deviation (RSD) of this method, obtained for nine determination of 0.03 µg·mL^−1^ of As, Bi, Cd, Hg, Pb and Ti, were 1.2%, 0.91%, 1.63%, 1.1%, 1.9%, and 1.57%, respectively. A comparison of the analytical performance in the present work with those reported in the literature is given in Table 7.

### 3.8. Analytical Ions in Real Sample

The preconcentration method proposed was applied to inductively coupled plasma atomic emission spectroscopy determination of analyte ions from skin whitening cosmetics purchased from the market in Saudi Arabia. All the measurements were run in three replicates for the samples and standard solutions. The analytical results concentration µg·mL^−1^ with standard deviation SD of three replicates are given in Table 8.

**Table 7:** Comparative data of the analytical performance with those in the literature.

Adsorbent	Method	Element	D. L. (ng·mL^−1^)	RSD (%)	Reference
Multiwalled carbon nanotubes	ICP-AES	Cd, Pb	0.30, 1.80	1.63, 1.90	This work
Amberlite XAD-4 resin coated with dithiocarbamates	ICP-AES	Cd, Pb	0.70, 1.70	2.56, 3.03	[14]
Multiwalled carbon nanotubes	AAS	Pb	8.00	2.50	[11]
Amberlite XAD copolymer resins	FAAS	Cd, Pb	1.80, 6.31	0.14–20.36	[15]
Multiwalled carbon nanotubes	FAAS	Pb	1.00	4.62	[10]
modified silica gel	ICP-AES	Pb	1.23	3.50	[16]
modified Activated Carbon	ICP-AES	Pb	0.36	1.90	[17]

**Table 8:** Heavy metals content in skin whitening cosmetic products (µg·g^−1^).

n	As concentration (* mean ± #SD)	Bi concentration (* mean ± #SD)	Cd concentration (* mean ± #SD)	Hg concentration (* mean ± #SD)	Pb concentration (* mean ± #SD)	Ti concentration (* mean ± #SD)
1	12.12 ± 0.04	1.45 ± 0.02	0.675 ± 0.008	1.70 ± 0.018	21.36 ± 0.150	11.27 ± 0.008
2	2.40 ± 0.05	273.45 ± 8.00	0.180 ± 0.007	BDL	252.00 ± 7.500	1,086.00 ± 15.00
3	4.49 ± 0.04	2.68 ± 0.03	BDL	BDL	219.16 ± 1.250	2.52 ± 0.080
4	1.28 ± 0.02	4.39± 0.01	1.100± 0.010	2.31 ± 0.007	102.30 ± 0.200	86.04 ± 0.470
5	3.15 ± 0.07	BDL	0.600 ± 0.007	9.66 ± 0.080	445.50 ± 2.250	1,025.20 ± 150
6	6.80 ± 0.09	BDL	BDL	22.78 ± 0.085	280.80 ± 1.610	204.10 ± 1.700
7	2.89 ± 0.08	11,842.50 ± 30.00	0.189 ± 0.009	2,745.00 ± 8.100	78.60 ± 0.150	56.30 ± 0.240
8	8.25 ± 0.14	2.12 ± 0.024	0.555 ± 0.008	24.03 ± 0.150	386.80 ± 3.750	11.25 ± 0.150
9	3.10 ± 0.03	BDL	0.320 ± 0.002	8.500 ± 0.052	279.90 ± 1.800	856.80 ± 4.000
10	3.09 ± 0.06	BDL	0.832 ± 0.008	10.21 ± 0.160	693.00 ± 0.520	299.60 ± 1.050
11	0.84 ± 0.01	2.25 ± 0.016	5.220 ± 0.026	1.00 ± 0.036	5.52 ± 0.046	2.78 ± 0.044
12	2.78 ± 0.09	59.43 ± 0.216	BDL	1.289 ± 0.018	109.50 ± 0.180	110.43 ± 0.540
13	11.71 ± 0.06	2.02 ± 0.052	0.500 ± 0.002	BDL	632.60 ± 2.250	3.33 ± 0.037
14	2.37 ± 0.01	0.68 ± 0.018	0.720± 0.003	1.77 ± 0.022	40.50 ± 1.280	3.53 ± 0.025
15	1.98 ± 0.03	BDL	0.171 ± 0.009	29.63 ± 0.160	288.45 ± 1.350	1,865.60 ± 19.200
16	BDL	BDL	0.720 ± 0.008	BDL	8.00 ± 0.080	3.73 ± 0.016
17	6.14 ± 0.07	1,959.20 ± 5.600	0.243 ± 0.001	2.10 ± 0.008	BDL	2.62 ± 0.016
18	0.34 ± 0.01	1.83 ± 0.025	0.76 ± 0.001	BDL	5.52 ± 0.170	7.65 ± 0.086
19	15.36 ± 0.12	13.81 ± 0.400	0.825 ± 0.007	1.95 ± 0.015	6.75 ± 0.050	2.90 ± 0.025
20	9.87 ± 0.07	BDL	2.060 ± 0.007	BDL	238.80 ± 0.900	160.80 ± 1.760
21	8.08 ± 0.05	266.40 ± 5.060	0.409 ± 0.010	417.16 ± 9.450	299.20 ± 1.680	728.96 ± 4.460
22	5.78 ± 0.12	383.00 ± 3.250	0.330 ± 0.010	4.34 ± 0.090	9.87 ± 0.197	310.00 ± 2.550
23	1.68 ± 0.04	BDL	0.500 ± 0.010	3.33 ± 0.033	21.56 ± 0.1950	228.10 ± 2.500
24	1.44 ± 0.01	BDL	0.150 ± 0.015	BDL	794.25 ± 9.750	841.20 ±6.000
25	14.76 ± 0.10	47.06 ± 2.740	0.082 ± 0001	2.60 ± 0.037	313.00 ± 1.700	43.74 ± 1.560
26	7.81 ± 0.05	847.50 ± 8.900	0.831 ± 0.023	BDL	106.30 ±1.300	678.30 ± 7.120
27	8.52 ± 0.15	BDL	0.520 ± 0.009	BDL	9.60 ± 0.312	2,749.00 ± 53.130
28	1.99 ± 0.03	BDL	0.328 ± 0.008	16.49 ± 0.024	114.70 ± 0.560	2,262.40 ± 9.600
29	5.54 ± 0.16	BDL	BDL	151.95 ± 0.450	BDL	37.80 ± 0.075
30	2.41 ± 0.03	59.95 ± 0.140	0.120 ± 0.001	9.68 ± 0.080	196.92 ± 0.490	241.10 ± 1.680
31	8.70 ± 0.05	BDL	0.232 ± 0020	BDL	116.70 ± 0.150	652.50 ± 3.370
32	2.18 ± 0.09	BDL	0.285 ± 0.008	0.63 ± 0.020	23.50 ± 0.220	352.05 ± 6.520
33	3.55 ± 0.08	BDL	BDL	5.21± 0.100	141.64 ± 0.440	437.48 ± 2.960
34	2.06 ± 0.020	0.61 ± 0.020	0.090 ± 0.001	9.39 ± 0.060	3.37 ± 0.010	9.00 ± 0.075

* mean concentration for three replicate; # standard deviation; N = 3 BDL: below the detection limit.

The results of heavy metals in the skin whitening cosmetics are shown in Table 8. Arsenic was found in all samples, except sample No. 16. It’s concentration varied from 0.34 to 15.36 µg·g^−1^. Sample 19 had the highest arsenic concentration and sample 18 had the lowest. According to the World Health Organization (WHO), the heavy metals concentration of herbal medicines must definitely be controlled, but the WHO is silent regarding the maximum permissible limits of heavy metals in herbal cosmetics [8]. According to the WHO, the permissible limit for arsenic in herbal preparations is 10 µg·mL^−1^. In that way, four samples were found to contain arsenic concentrations above the permissible limit. Bismuth was found in 19 samples and it’s concentrations varied from 0.615 to 11,842.5 µg·g^−1^. Sample 7 had the highest bismuth concentration and sample 34 had the lowest. Bismuth was also found below the detectable limit in 15 samples. Cadmium was found in 29 samples and it’s concentration varied from 0.09 to 5.22 µg·g^−1^. Sample 11 had the highest cadmium concentration and sample 34 had the lowest. Cadmium was also found below the detectable limit in three samples. According to the WHO, the permissible limit for cadmium is 0.3 µg·mL^−1^ in herbal preparations, thus 19 samples were found to contain arsenic concentrations above the permissible limit. Mercury was found in 24 samples. It’s concentration varied from 0.637 to 2,745 µg·g^−1^. These values are extremely high and represent a serious health hazard. Sample 7 had the highest mercury concentration and sample 32 had the lowest. Mercury was found below the detectable limit in 10 samples. According to the World Health Organization (WHO), the permissible limit for mercury in cosmetic is 1.0 µg·mL^−1^. In this work, 22 samples were found to contain mercury concentrations above the permissible limit. Lead was found in all samples except for two, and it’s concentration varied from 3.37 to 794.25 µg·g^−1^. Sample 24 had the highest lead concentration and sample 34 had the lowest. Lead was also found below the detectable limit in two samples. According to the WHO, the permissible limit for lead is 10.0 µg·mL^−1^. We were found 26 samples above the permissible limit and unfortunately, all the skin whitening cosmetic products were found to contain lead concentrations higher than the permissible limit. Titanium was found in all samples. It’s concentration varied from 2.52 to 2,749 µg·g^−1^. Sample 27 had the highest titanium concentration and sample 3 had the lowest. Were also compared to results obtained with the results of previous studies as shown in Table 9.

**Table 9:** Compare the results ofthe study withprevious studies.

Heavy metal	Concentration range (µg·g^−1^)	Ref.
As, Bi, Cd,	(0.34–15.36), (0.615–1,1842.5), (0.09–5.2),	This work
Hg, Pb, Ti	(0.637–2,745), (3.37–794.25), (2.52–2749)	
Hg	(878–36,000)	[18]
Hg	(2.46–23,222)	[19]
Hg	(660–57,000)	[20]
Pb	(87–123)	[21]
Hg, As, Cd, Pb	(0.04–2.183), (0.690–3.683), (0.625–1.875), (1.470–33.1)	[7]
Hg, As	(5,700–126,000), (522–161,600)	[22]

## 4. Conclusions

The use of multiwalled carbon nanotubes as solid phase extraction sorbent for the preconcentration of the heavy metals prior to their determination by inductively coupled plasma atomic emission spectrometry (ICP-AES) analysis, proved to be a very powerful, advanced, rapid and precise technique for the analysis of heavy metals in skin whitening cosmetic creams. Results obtained from the certified reference material of NIST SRM 1570a spinach leaves using the proposed microwave digestion method and preconcentration procedure show very good recoveries (93.3–102.9%) of several metals in cosmetics and thus this method can be easily used for routine analysis of such samples in laboratories.
